# New arguments in the discussion about the nature of picobirnaviruses

**DOI:** 10.18699/vjgb-26-13

**Published:** 2026-03

**Authors:** A.Yu. Kashnikov, N.V. Epifanova, N.A. Novikova

**Affiliations:** I.N. Blokhina Nizhny Novgorod Research Institute of Epidemiology and Microbiology, Nizhny Novgorod, Russia; I.N. Blokhina Nizhny Novgorod Research Institute of Epidemiology and Microbiology, Nizhny Novgorod, Russia; I.N. Blokhina Nizhny Novgorod Research Institute of Epidemiology and Microbiology, Nizhny Novgorod, Russia

**Keywords:** picobirnavirus, genome segment, host cell, mitochondrial genetic code, reassortment, пикобирнавирус, сегмент генома, клетка-хозяин, митохондриальный генетический код, реассортация

## Abstract

Picobirnaviruses (PBVs), members of the Picobirnaviridae family, are found in a wide range of hosts, including eukaryotes (both higher and lower), fungi, and bacteria. However, scientists are unsure about their “true master” or primary host. While often found in animals, including cases of gastroenteritis, they are also detected in environmental samples and have shown genetic links to bacterial and fungal viruses. The lack of a reliable cell culture or animal model for PBV propagation further complicates determining their host specificity. Due to the discovery of prokaryotic regions (motifs) in segments of the PBV genome, it was suggested that their hosts are prokaryotic. However, even this discovery did not pin one specific host to PBVs; since then PBV-like genomes not characteristic of the studied PBV strains, with a mitochondrial genetic code characteristic of lower eukaryotes (molds and invertebrates), were discovered. And recently, a new version of the origin of PBVs from vertebrate viruses and fungi has appeared, denying their phage nature. To understand the nature of genetically diverse PBV strains detected in different organisms, researchers were guided by information about the presence of motifs specific to the viral family in the genome, the genetic code used, and the method of distribution. Recent research suggests that PBVs, previously thought to have a vertebrate origin, may have also evolved from fungal sources denying their phage nature. Some PBV-like sequences have been found to utilize the fungal mitochondrial genetic code, indicating a possible fungal origin or a close relationship with fungal viruses like mitoviruses. This discovery challenges the previously held view of PBVs as exclusively vertebrate viruses and suggests a more complex evolutionary history. The information available today inspires confidence in the imminent conclusion of the ongoing discussion about the possible PBV hosts. In particular, a hypothesis has recently emerged demonstrating a possible mechanism for the replacement of the genetic code in RNA viruses, which makes it possible to explain the origin of PBV forms with the mitochondrial genetic code capable of reproduction in cells of lower eukaryotes using the example of phages. However, an evolutionarily deterministic model demonstrating the path of PBV formation with the genetic code of mold and invertebrate cells has not yet been presented. According to the authors of this review, this evolutionary path is due to the endosymbiotic relationships between the putative PBV hosts, contributing to the horizontal virus spread. The purpose of this review article is to attempt to describe a possible path of formation from the ancestral PBV form and its derived evolutionary forms, some of which inherited a genome with a prokaryotic motif and a standard genetic code, while others acquired a non-standard form of the genome with the code of lower eukaryotes. This review article focuses on the leading role of horizontal transmission in the formation of non-standard intermediate PBV forms.

## Abstract (ru)

Пикобирнавирусы (ПБВ) из семейства Picobirnaviridae находят у самых разных хозяев – высших и низших эукариот, а также в грибах и бактериях. Однако среди ученых в отношении ПБВ на сегодняшний день нет однозначного понимания, кто является их истинным хозяином. Принадлежность ПБВ к вирусам высших эукариот не доказана, поскольку не подобраны ни культура животных клеток для их размножения, ни животное-гнотобионт. В связи с обнаружением прокариотических участков (мотивов) в сегментах генома ПБВ было высказано предположение о прокариотической природе их хозяев. Однако и это открытие не закрепило одного конкретного хозяина за ПБВ, так как затем были обнаружены ПБВ-подобные геномы, не характерные для изученных штаммов ПБВ, – с митохондриальным генетическим кодом, свойственным низшим эукариотам (плесени и беспозвоночным). А недавно появилась новая версия происхождения ПБВ от вирусов позвоночных и грибов, отрицающая их фаговую природу. Для понимания природы генетически разнородных штаммов ПБВ, обнаруженных у разных организмов, исследователи руководствовались информацией о присутствии специфических для вирусного семейства мотивов в геноме, используемом генетическом коде и способе распространения. Существующая в настоящее время информация вселяет уверенность в скором завершении продолжающейся дискуссии о возможных хозяевах ПБВ. В частности, недавно появилась гипотеза, демонстрирующая вероятный механизм замены генетического кода у РНК-вирусов, которая позволяет объяснить происхождение форм ПБВ с митохондриальным генетическим кодом, способных к репродукции в клетках низших эукариот на примере фагов. Однако еще не представлена эволюционно детерминированная модель, демонстрирующая путь формирования ПБВ с генетическим кодом клеток плесени и беспозвоночных. Этот эволюционный путь в представлении авторов данного обзора обусловлен эндосимбиотическими отношениями между предполагаемыми хозяевами ПБВ, способствующими горизонтальному распространению вируса. Цель нашей статьи – попытка описания возможного пути формирования из предковой формы ПБВ ее производных эволюционных форм, одни из которых унаследовали геном с прокариотическим мотивом и стандартным генетическим кодом, а другие обрели нестандартную форму генома с кодом низших эукариот. В статье делается акцент на ведущей роли горизонтальной передачи в формировании нестандартных промежуточных форм пикобирнавирусов

## Discussion about the nature of picobirnaviruses

Picobirnavirus (PBV) is the only genus in the Picobirnaviridae family placed under the order “Diplornavirales” (Reddy et al., 2023). PBV particles with a diameter of 33–37 nm with a single-layer protein shell have icosahedral symmetry. There is no lipoprotein membrane. PBV genome consists of two dsRNA segments (Delmas et al., 2019). The larger segment (2.4–2.6 kb) (segment 1) encodes a capsid protein and a protein of unknown function, and the smaller segment (1.5–1.9 kb) (segment 2) encodes RNA-dependent RNA polymerase (RdRp of the Pfam family RdRp_1). Based on the RdRp sequence, PBVs are grouped into genogroups, with genogroup I (GI) and genogroup II (GII) being the most common (Malik et al., 2014; Reddy et al., 2023).

Initially, PBVs were detected in the intestinal contents and in the respiratory tract of higher eukaryotes (Pereira et al., 1988; Novikova et al., 2003; Delmas et al., 2019; Kumar et al., 2020; Ghosh, Malik, 2021). In symptomatic infections, PBVs were often detected in the intestines of animals and humans in association with viruses, the pathogenicity of which has been established. However, these viruses are detected both in symptomatic and asymptomatic cases. For this reason, PBVs have traditionally been considered opportunistic intestinal viruses of mammals and birds (Shi et al., 2016; Delmas et al., 2019; Kumar et al., 2020). Since PBVs could not be successfully propagated in mammalian or gnotobiont cell cultures, researchers had doubts about the association of PBVs pre- sent in the intestine with animal disease (Ghosh, Malik, 2021; Sadiq et al., 2024). PBVs have been found in invertebrates (mollusks, arthropods, insects) (Shi et al., 2016), and more recent studies have shown that these viruses are likely to infect prokaryotic or fungal host cells (Adriaenssens et al., 2018; Boros et al., 2018; Krishnamurthy, Wang, 2018; Yinda et al., 2018; Kleymann et al., 2020).

Currently, three alternative versions about the nature of PBV hosts are being discussed. According to the first version, the PBV hosts are cells of higher eukaryotes (animals). According to the second version, PBVs may be prokaryotic viruses. This assumption was associated with the discovery of regions (motifs) characteristic of prokaryotic viruses in the PBV genome. According to this version, since PBV-like nucleotide sequences are often found in the animal stool samples, they may be related not to the cells of these animals, but to the presence of the corresponding bacteria in the intestinal microbiome. However, atypical PBV-like genomes with prokaryotic motifs, but with a mitochondrial genetic code characteristic of lower eukaryotes, such as molds and invertebrates, were subsequently isolated from the animal intestinal microbiome (Yinda et al., 2018; Kleymann et al., 2020). So, a third version appeared according to which in addition to prokaryotic cells, some PBVs isolated from the animal intestinal contents can infect the mitochondria of fungi or invertebrates (Shi et al., 2016; Ghosh, Malik, 2021).

There is still no clear answer on the debated issue – which organism(s) are the PBV hosts – although over the years alternative points of view about the nature of PBVs have been supplemented with new arguments in their support. In particular, recently, in favor of the hypothesis denying the phage nature of PBVs, a new idea has emerged about the parallel evolution of PBVs from two different ancestors that were parasites of fungi and vertebrates (Perez et al., 2023).

## The version according to which the PBV ancestors are vertebrate and fungal viruses

This version describes an immune response in a vertebrate host based on the recognition of a pathogen, specifically a virus called PBV. The immune system reacts when it detects PBVs containing the standard genetic code, but it does not mount a response when it encounters PBVs with an alternative genetic code, specifically one used by a fungus. The version provides two different evolutionary development models of the PBV amily, which was formed from three different ancestral lines. According to L.J. Perez et al. (2023), the ancestral PBV lineages (PBV-R1 and PBV-R3) are descended from vertebrate reoviruses (because they provide immunity in the vertebrate in the intestine of which they are detected), and the ancestral PBV-R2 line is descended from fungal partiviruses (immunity in the animal in which they were detected not observed).

**Fig. 1.**
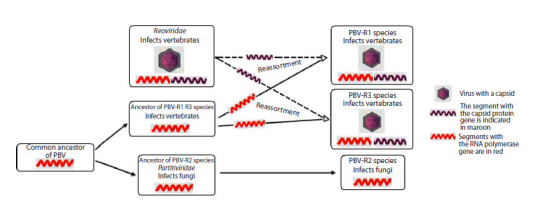
The first evolutionary development model of the PBV family according to L.J. Perez et al. (2023).

According to the first evolutionary development model, at first the common PBV ancestor, containing only the RdRp gene, split into two species. One of them later split into two more species with the simultaneous evolutionary acquisition of a capsid by both species as a result of the reassortment of segments of their genomes with segments of the genome of their common ancestor belonging to the family Reoviridae. As a result, two encapsidated ancestral PBV species were located on the same branch of the phylogenetic tree. Together with the previously separated PBV-R2 species, three PBV ancestral species (lines) evolving in parallel were formed during further diversification (Fig. 1).

**Fig. 2.**
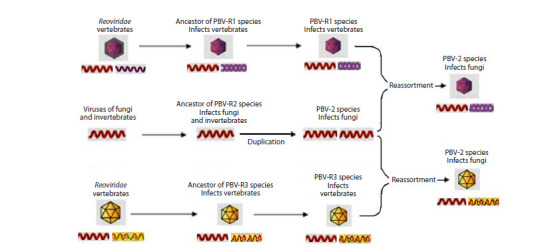
The second evolutionary development model of the PBV family according to L.J. Perez et al. (2023). Segments with capsid protein genes are indicated in maroon and yellow; segments with RNA polymerase genes are indicated in red.

According to the second evolutionary development model, three PBV-like ancestral species containing the RdRp gene descended from different ancestors, while two species possessed a capsid from the moment of their appearance and, as in the first model, formed a common branch presumably evolving from vertebrate reoviruses (Fig. 2).

The third fungal species (PBV-R2), initially devoid of a capsid, acquired a segment with the capsid gene later as a result of duplication and reassortment of genome segments with one of the two PBV species with capsid infecting vertebrates, according to the mechanism described (Luo et al., 2018). This ancestral species initially resembled mitoviruses with a mitochondrial code, similar to PBV-like strains found by C.K. Yindaet al. (2018) and A. Kleymann et al. (2020), and later it was located on the same branch as the Partitiviridae family, which infects non-chordate eukaryotes (fungi and invertebrates).

The parallel evolution of these three PBV ancestors under similar living conditions could lead to the formation of similar and at the same time genetically diversified representatives of the Picobirnaviridae family (Perez et al., 2023). Like the version about the phage nature of PBVs, this version has the right to exist. But the proponents of the version that defends the phage nature of PBVs also present new arguments in favor of their point of view.

## The point of view according to which PBVs can infect prokaryotic cells

Proponents of the hypothesis suggesting that prokaryotic cells may be the PBV hosts cite the following arguments in support of the phage nature of PBVs

1. The PBV genome is enriched (compared to eukaryotic viruses) with prokaryotic motifs – Shine–Dalgarno sequences (SD, “GGAGG” hexamer) similar to the genomes of bacteriophages of the Cystoviridae family (Ghosh, Malik, 2021), in which this motif occurs even less frequently (Krishnamurthy, Wang, 2018). For example, a recent study by S. Sadiq et al. (2024) showed that the PBV genome was enriched with the SD motif in 83–85 % of ORF (open reading frames) in the Picobirnavirus genomes, which exceeds the enrichment of the genomes of the RNA bacteriophages Leviviricetes and Cystoviridae with this motif.

2. PBVs are detected almost exclusively in animal stool samples and still cannot be cultured in any eukaryotic cell lines. This may also mean that PBVs are actually bacterial viruses that are part of the animal intestinal microflora or components of their food (Adriaenssens et al., 2018; Delmas et al., 2019; Bell et al., 2020; Guajardo-Leiva et al., 2020; Ghosh, Malik, 2021; Knox et al., 2023; Sadiq et al., 2024). This assumption is consistent with the fact that their closest relatives, representatives of the Partitiviridae family, infecting plants, fungi and protozoa (Vainio et al., 2018), are also found in the animal intestinal microflora (Chen et al., 2021) and in the microbiome of invertebrates (Shi et al., 2016; Le Lay et al., 2020).

3. The arrangement of genome segments in typical PBVs is observed in a single particle, as in bacterial viruses, while in most fungal viruses with a segmented genome, the segments are encapsulated separately (Luque et al., 2018).

4. The assumption of opponents of the phage nature of PBVs (Perez et al., 2023) that viruses forming immunity in infected animals (or humans) should belong to eukaryotic viruses cannot unequivocally support the version of the eukaryotic nature of PBV hosts, since it has been established that host immune responses can also occur against bacterial viruses (Dabrowska et al., 2005; Górski et al., 2006). Consequently, PBVs can elicit an immune response not to infection of the animal’s own cells, but of the bacterial cells that make up its microbiome (Ghosh, Malik, 2021).

5. Identification of a protein unique to a virus is a key step in determining its specific host, since the presence of bacteriolytic properties in its owner can convincingly indicate that this virus is a bacteriophage (Gan, Wang, 2023; Kashnikov et al., 2023). As it turned out, a protein with a lysing function, which lyses Escherichia coli, is present in the PBV capsid, and such proteins are encoded only by the genes of two known families of RNA phages, Leviviridae and Cystoviridae (Cai et al., 2021). However, perhaps not all, but only some PBVs encode bacteriolysins (Gan, Wang, 2023).

6. A recent study by S. Sadiq et al. (2024) can also confirm the assumption about the phage nature of PBVs. In this study, based on clustering, seven PBV clusters were identified in the microbiome, which could only be hosted by cells of different microbes, since there was no phylogenetic grouping by PBVs belonging to host animals with high genetic variability of PBVs. The lack of grouping between the virus and the host indicates a phylogenetic discrepancy between them (Duraisamy et al., 2018; Woo et al., 2019; Mahar et al., 2020). Based on this, S. Sadiq et al. (2024) suggested that PBVs infect various microbial organisms associated with vertebrates and invertebrates through their diet and habitat.

Based on these observations, proponents of the phage nature of PBVs believe that there could not have been a wide variety of PBVs if their hosts were only mammals, birds, and invertebrates (Sadiq et al., 2024). They suggest that clustering associated with bacteria is confirmed by the high rate of spread of PBVs among animals (even higher than in any other family of RNA viruses). This rate of spread is more consistent with PBVs belonging to prokaryotic hosts, with their extensive interspecific transmission. In addition, proponents of the phage nature of PBVs guess that the preservation of prokaryotic SD sequences is impossible in viruses infecting fungi (Ullah et al., 2022; Wang, 2022). This argument is supported by the probable ability of PBVs, like some phages, to change their genetic code.

**Replacement of the genetic code as a trend in phage evolution**

In the genomes of some microorganisms, it is possible to replace the standard genetic code with an alternative (mitochondrial) one. This replacement, according to Y. Shulgina and S.R. Eddy (2021), is associated with a decrease in the GC base content during evolution due to genome reduction in microorganisms, which may be due to their parasitic mode of existence – endosymbiosis (McCutcheon, Moran, 2011). In particular, a decrease in the number of GC bases in the genome of organisms leads to a decrease in the proportion of the TGA stop codon (with an increased probability of reassignment of this codon) (Korkmaz et al., 2014).

The discovery of alternative genetic codes in the mitochondrial genome of mold fungi and invertebrates, in bacteria and archaea demonstrates the genetic code’s ability to evolve (Shackelton, Holmes, 2008; Kollmar, Mühlhausen, 2017; Shulgina, Eddy, 2021). The replacement of the genetic code with an alternative one in the mitochondria of eukaryotic cells, bacteria, and archaea could presumably be related to their drive to escape from bacteriophages, which initially used the same code as bacteria (Bender et al., 2008).

Bacteriophages are also capable of changing their genetic code when it becomes necessary to deceive the protective antiviral systems of the host bacterium. For example, it was found that the genetic code in the genome of some phages isolated from baboon stool samples corresponds to an “alternative” genetic code of the genomes of Bacilli bacteria (Al-Shayeb et al., 2020). A study by N. Yutin et al. (2021) reports on the replacement (recoding) of stop codons (TAGS) in crAssphage DNA bacteriophages during translation to a codon encoding the amino acid glutamine. The reassignment of the standard TGA stop codon to an alternative one encoding the amino acid tryptophan, corresponding to the code of fungal and invertebrate mitochondria, was observed in some PBV-like genomes by C.K. Yinda et al. (2018), who first discovered this phenomenon in PBVs.

According to D. Wang (2022), the tendency to change the PBV genetic code, as in bacteriophages, is probably related to the ability to read or recode the TGA stop codon during translation, regardless of the taxonomic affiliation of the cells they infect (since phages probably target mitochondria in eukaryotic cells, given their bacterial origin). It is possible that some PBV strains, like bacteriophages, once in a fungal cell, are able to capture the suppressor transfer RNA (tRNA) encoded by the host, or somehow disrupt the host’s translation mechanism (Wang, 2022).

According to S.L. Peters et al. (2022), some phages probably use bacterial ribosomes using both standard and alternative codes to translate their proteins. At the same time, the reassignment of normal TAG and TGA stop codons to translation to glutamine and tryptophan is especially common in phages infecting gram-positive bacteria such as Firmicutes and Bacteroidetes (Peters et al., 2022). Firmicutes, known for their low GC content in the genome (less than 50 %), probably related to their parasitic mode of existence, “endosymbiosis”, often experience a change of the standard code to an alternative one. It was previously noted that Firmicutes are hypothetically most suitable as PBV hosts, which presumably may be phages (Krishnamurthy, Wang, 2018). It is possible that atypical PBV strains with an “alternative” fungal genetic code (Yinda et al., 2018; Kleymann et al., 2020), like phages, were transcoded during translation (Peters et al., 2022) following their host Clostridial Firmicutes, which parasitizes the fungal cell.

Thus, it remains likely that PBVs using the fungal mitochondrial code are bacteriophages in which the code replacement is due to evolution associated with the prokaryotic host. Moreover, E.V. Koonin et al. (2020) suggest that PBVs, which were previously considered animal viruses, are exclusively bacterial viruses, in contrast to the phylogenetically close family Partitiviridae, which includes eukaryotic and, according to recent data, bacterial viruses, since they, like PBVs, have bacteriolysins. According to U. Neri et al. (2022), the Picobirnaviridae family may represent the third clade of RNA bacteriophages together with the Leviviridae and Cystoviridae families.

## Can cells of mold fungi and invertebrates be PBV hosts?

Some arguments by proponents of the phage nature of PBVs cast doubt on the existence of forms of PBVs capable of infecting cells of lower eukaryotes. However, the fact that atypical PBV forms have been identified, the genetic code of which corresponds to viruses of mold fungi and invertebrates, suggests that not all PBVs encode bacteriolysins and, probably, in some PBV forms, like Mitoviridae or Partitiviridae, cells of lower eukaryotes can be hosts instead of bacterial cells (Luo et al., 2018; Shi et al., 2018; Yinda et al., 2018; Ghosh, Malik, 2021; Ullah et al., 2022; Reddy et al., 2023). The prerequisites for this possibility are: the origin of eukaryotic RNA viruses from +RNA phages (Wolf et al., 2018) and their predisposition to horizontal transmission (Son et al., 2015; Dolja, Koonin, 2018).

**The origin of eukaryotic RNA viruses from +RNA phages as evidence of the ability of viruses to change their taxonomic nature**

**Fig. 3.**
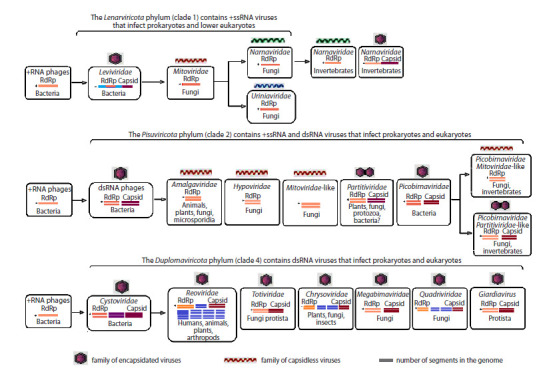
The evolutionary development model of viral families originating from prokaryotic RNA viruses, according to a version supported by researchers (Koonin et al., 2015; Wolf et al., 2018; Ghosh, Malik, 2021; Sadiq et al., 2022).

The currently available results of phylogenetic studies indicate the existence of a relationship between the families of eukaryotic and prokaryotic RNA viruses and their common origin (Fig. 3) (Dolja, Koonin, 2018; Wolf et al., 2018). In particular, according to E.V. Koonin et al. (2015), bacteriophages of the Cystoviridae family are evolutionarily related to the Reoviridae family (eukaryotic viruses) and may be direct ancestors of the Picobirnaviridae family.

Based on the analysis of RNA-dependent RNA polymerase, the relationship between single-stranded RNA bacteriophages of the Leviviridae family from the Leviricetes class and mitoviruses has been proven (Wang, 2022). According to researchers, the families of Lenarviricota-type RNA viruses (Mitoviridae, Narnaviridae, Urmiaviridae) are evolutionarily transitional forms from RNA bacteriophages to early RNA viruses infecting lower eukaryotes (Wolf et al., 2018; Sadiq et al., 2022). According to the researchers, the evolutionary transition of prokaryotic viruses to forms of RNA viruses infecting eukaryotes occurred approximately 1.45 billion years ago due to the transition of α-proteobacteria to an endosymbiotic mode of existence. At the same time, forms that lost their capsid due to this evolutionary transition, such as Mitoviridae, switched to reproduction in mitochondria, and later, like Narnaviridae, entered the cytosol of the cell (Dolja, Koonin, 2018; Wolf et al., 2018).

Y.I. Wolf et al. (2018) believe that different groups of RNA viruses could have evolved independently from prokaryotic +RNA viruses, since their RdRp genes are more similar to the RdRp genes of ancestral +RNA viruses than to the genes of other RNA viruses from parallel branches of the phylogenetic tree

**Role of the method of RNA virus spread in the formation of their taxonomic affiliation**

Phylogenetic studies have shown that RNA viruses belonging to the same family can infect hosts from different taxa, including fungi, plants, animals, and protozoa (Son et al., 2015). Moreover, the vast majority of RNA virus families infect unicellular eukaryotes using alternative genetic codes corresponding to these organisms (Neri et al., 2022). These studies are consistent with the “Ancient Coevolution Hypothesis” (Pearson et al., 2009), which states that viruses can migrate from a host belonging to one taxonomic category to a host from another taxonomic category. A similar relationship with organisms from different taxa is observed, for example, in RNA viruses of the families Partitiviridae, Reoviridae, Amalgaviridae, Totiviridae, and Chrysoviridae. The members of the Lenarviricota, Duplornaviricota, and Pisuviricota RNA viruses (PBVs belong to Pisuviricota) are very diverse, distributed in almost any environment, and associated with a wide range of hosts, encompassing bacteria, protozoa, fungi, and plants (Dolja, Koonin, 2018; Sadiq et al., 2022).

According to the “Hypothesis of ancient coevolution”, capsidless mitovirus-like PBV forms, like mitoviruses, may represent a transitional evolutionary form from the ancestral +RNA prokaryotic viruses to the simplest eukaryotic viruses (Wolf et al., 2018). Having inherited from its ancestor, the prokaryotic virus, the RdRp gene with a prokaryotic motif and specific motifs (DFXKFD, SGSGGT, and GDD), this form, upon transition to a new host, the fungus, could acquire its genetic code, which its original prokaryotic host could borrow while in an endosymbiotic relationship with the fungus.

Endosymbiosis (symbiogenesis) is a type of symbiosis when one of the partners lives inside the other’s cell. In endosymbiosis, the larger of the partners is usually called the host, and the partner is an organism living inside its host, a parasite if it competitively affects its host, suppressing its reproduction. In many associations, parasitic microorganisms move to a permanent intracellular existence and are inherited. The loss of the capsid by the transitional PBV form (similar to Mitoviridae) required reproduction inside mitochondria or vacuoles (as in Narnaviridae) in order to avoid selective destruction by the cell protection system of its ds genome corresponding to the replicative form of the Mitoviridae genome formed at the intermediate stage of replication. In this case, the PBV-like form P16-366 characterized by the presence of a capsid protein (Yinda et al., 2018) can be considered as an evolutionarily more advanced transitional form, since the presence of a capsid protein in this PBV form does not require hiding its ds genome in the mitochondria of the fungus (Wolf et al., 2018).

A recent large-scale metagenomic study of eukaryotic +RNA cells may confirm the possibility of such an evolutionary scenario. +RNA viruses of the Narnaviridae family, which is one of the two known descendant families of +RNA phages hosted by fungi. This study revealed numerous narnalike genovariants among the representatives of Narnaviridae, both without the capsid protein gene and with the capsid gene, which were hosted by various invertebrates (Shi et al., 2016). In modern taxonomy, mito- and narna-like RNA viruses descending from bacteriophages with the fungal mitochondrial genetic code have received the status of a family and are defined as eukaryotic RNA viruses. Moreover, while at the family level the spectrum of RNA virus hosts can be wide, at the genus level this spectrum is usually limited and there are clear phylogenetic differences between genera infecting hosts from different taxa (Sadiq et al., 2024).According to Y.I. Wolf et al. (2018), the origin of PBVs or partiviruses is explained by reassortment between the genome segment of the RdRp virus with +ssRNA from the partivirus-picobirnavirus clade (with a mitovirus-like reproduction method) and the segment with the capsid gene of the virus with the dsRNA genome from the clade of cystoviruses, totiviruses and reoviruses.

Reassortment is a form of exchange of genetic information between viruses with a segmented genome. In viruses with a segmented genome, segment exchange is possible when two (or more) genetically distinct viruses simultaneously infect the same cell. The viruses between which reassortment occurs form a new species or strain of virus with different qualities and, in most cases, increased pathogenicity. Reassortment in nature most often occurs within a single species, but it can also occur within a single genus.

The possibility of reassortment between segments of viral genomes formed by dsRNA and +ssRNA can be explained by the participation in reassortment by +RNA viruses of segments at the stage of replicative intermediate forms of RNA (dsRNA). This variant of the origin of PBVs explains the fact that C.K. Yinda et al. (2018) identified PBV forms with and without capsid with an alternative genetic code, resembling fungal viruses by the method of reproduction.However, in accordance with the “theory of endosymbiosis”, capsidless PBV-like replicons with the fungal mitochondrial genetic code (P11-300, P11-378, P14-90, P15-218, WGML128211, M17A) discovered by (Yinda et al., 2018; Kleymann et al., 2020) can be considered not only as products of the exchange of homologous segments between families of related RNA viruses, but also as a result of symbiotic relationships.

According to the currently available data, PBV genomes with prokaryotic motifs and motifs of mold fungi and invertebrates have been found in the intestines of animals, as part of a single microbiota consisting of bacterial cells and protozoan eukaryotes. The detection of PBVs in the same microbiome with the genetic code of bacterial and mold fungal cells suggests the presence of endosymbiotic relationships between their hosts (Bruto et al., 2014). Recent studies examining the composition of the intestinal microbiota, including bacteria and fungi, indicate that there is a significant interdependence between fungi and bacteria (Li et al., 2022).

The existence of symbiotic relationships between taxonomically different RNA virus hosts allows the virus to overcome the barrier between symbiont cells by horizontal transfer with the possibility of further reproduction in the cells of a new host. An extended phylogenetic analysis proves the widespread horizontal transfer of RNA viruses between different hosts and its leading role in the evolution of these viruses (Dolja, Koonin, 2018). It has been established that bacteria are able to penetrate into the cytoplasm of fungi and stay there for a long time (https://www.pravda.ru/news/science/2024301-simbioz/). Moreover, horizontal transfer of genes or viruses, as a rule, is carried out from bacteria to fungi. In the opposite direction, the transfer of genes (viruses) is hardly possible (mycoviruses are transmitted by fungal cells exclusively vertically – from parents to offspring) (Bruto et al., 2014).

Firmicutes, the alleged PBV hosts (Krishnamurthy, Wang, 2018), being the most common microorganism in the intestines of animals and humans, could end up there along with fungal cells. There is evidence that some Firmicutes species, in particular Clostridial Firmicutes, competitively interact in the intestine with cells of the fungus Candida albicans, preventing the colonization of cells of higher eukaryotes by this fungus (Shulgina. Eddy, 2021). The parasitic mode of existence (endosymbiosis), which binds Firmicutes to the unicellular fungi C. albicans, allows horizontal transfer of viruses and genes between them (Taggart et al., 2023).

The discovery of PBV genomes with the genetic code of taxonomically different hosts also explains some general patterns in the evolution of dsRNA viruses, which the researchers noticed. This pattern is related to the ability of small dsRNA viruses with minimalistic genomes formed by a minimum number of segments encoding one RdRp protein (Narnaviridae) or two RdRPs and a capsid protein (Totiviridae, Partitiviridae, Picobirnaviridae) to perform non-specific horizontal distribution among taxonomically diverse hosts (Dolja, Koonin, 2018). For this reason, PBVs could probably also enter invertebrate cells, since invertebrates are particularly indiscriminate viral hosts.

Often, distantly related invertebrates can be hosts of the same group of viruses (Wolf et al., 2018). On the other hand, dsRNA viruses with the largest possible genome size, such as the Reoviridae family, are characterized by a much higher degree of host specificity, probably due to greater adaptation to it through the acquisition of genes involved in virus–host interactions (Dolja, Koonin, 2018).

It is believed that bacterial viruses cannot directly infect cells of higher eukaryotic organs, and they can enter these organs only by non-specific translocation with the help of bacteria in which they multiply (Dabrowska et al., 2005). It has only recently been reported that mammalian cells are able to directly internalize bacteriophages (Bichet et al., 2023) through macropinocytosis (non-specific internalization) and in rare cases through receptor-mediated endocytosis (specific internalization) (Bichet et al., 2023). However, to date, the ability of PBVs to penetrate animal cells has not been proven.

## Conclusion

Considering the sources at our disposal, which provide the molecular and genetic characteristics of the PBV strains discovered so far, it can be assumed that prokaryotes are most likely the hosts of naturally occurring PBV strains, and their transitional evolutionary forms may be present in unicellular eukaryotes (molds and invertebrates).

In favor of this assumption, we present the following arguments, which, as it seems to us, are the most significant.

**Arguments supporting the possibility of PBV reproduction in prokaryotic cells:**

• The PBV genome is enriched with SD sequences, which are inherent in prokaryotes (Krishnamurthy, Wang, 2018). • The presence of a bacteriolytic protein in PBVs can serve as a convincing argument that PBVs are bacteriophages (Gan, Wang, 2023). • The propensity of PBVs to genetic changes (characteristic of viruses with a segmented genome) is more characteristic of prokaryotic viruses. • Ubiquitous detection of PBVs in the intestines of animals of various levels of organization (vertebrates and invertebrates) and in wastewater (Ghosh, Malik, 2021); • Inability to cultivate PBVs in the laboratory or isolate them from animal tissue samples (Sadiq et al., 2024). • More frequent interspecific transmission of PBVs than animal RNA viruses from any other family (Sadiq et al., 2024). • The phenomenon of reassignment during translation of TAG and TGA stop codons to alternative ones (encoding amino acids glutamine and tryptophan) is especially common in phages infecting Firmicutes and Bacteroidetes (Peters et al., 2022).

**Arguments pointing to the possibility of the existence of some transitional evolutionary PBV forms in molds and invertebrates:**

• The common origin of the families of prokaryotic and eukaryotic RNA viruses allows for the existence of a wide range of RNA virus hosts related to different taxa at the family level (Wolf et al., 2018). • The endosymbiotic relationship between Firmicutes (putative PBV hosts) and C. albicans fungi (Peters et al., 2022), occupying the same ecological niche (animal or human intestines), suggests the possibility of horizontal transfer of genes and viruses between them (Shulgina, Eddy, 2021). • The endosymbiotic relationship between Firmicutes and C. albicansis being the reason for the PBV code replacement following its host Firmicutes (Bender et al., 2008). • The mechanism by which PBV genetic code changes (Wang, 2022) may demonstrate not only the ability of phages to reproduce in cells regardless of their taxonomic affiliation, but also a possible pathway for the formation of transitional evolutionary PBV forms found in the cells of some lower eukaryotes. • Only some PBVs have proteins with bacteriolytic functionin the capsid (Wang, 2022), as well as in the closely related family Partitiviridae, the representatives of which belong to fungal viruses (Neri et al., 2022).

These observations confirm the possibility of the existence of separate PBV forms, taxonomically related to both bacteria and unicellular eukaryotes – mold fungi and invertebrates. Therefore, as D. Wang et al. (2022) rightly noted, the final conclusions regarding the true host(s) of the identified PBVs cannot be generalized at the family level, but require studies of the whole diversity of PBVs in order to determine the taxonomic affiliation of the entire spectrum of their hosts.

## Conflict of interest

The authors declare no conflict of interest.
